# Whole-Genome Sequence of the *Trypoxylus dichotomus* Japanese rhinoceros beetle

**DOI:** 10.17912/micropub.biology.000487

**Published:** 2021-10-11

**Authors:** Norichika Ogata

**Affiliations:** 1 Nihon BioData Corporation

## Abstract

The draft whole-genome sequence of the Japanese rhinoceros beetle, *Trypoxylus dichotomus* was obtained using long-read PacBio sequence technology. The final assembled genome consisted of 739 Mbp in 2,347 contigs, with 24.5× mean coverage and a G+C content of 35.99%.

**Figure 1. Repeat sequences in the genomes of 10 beetle species:**
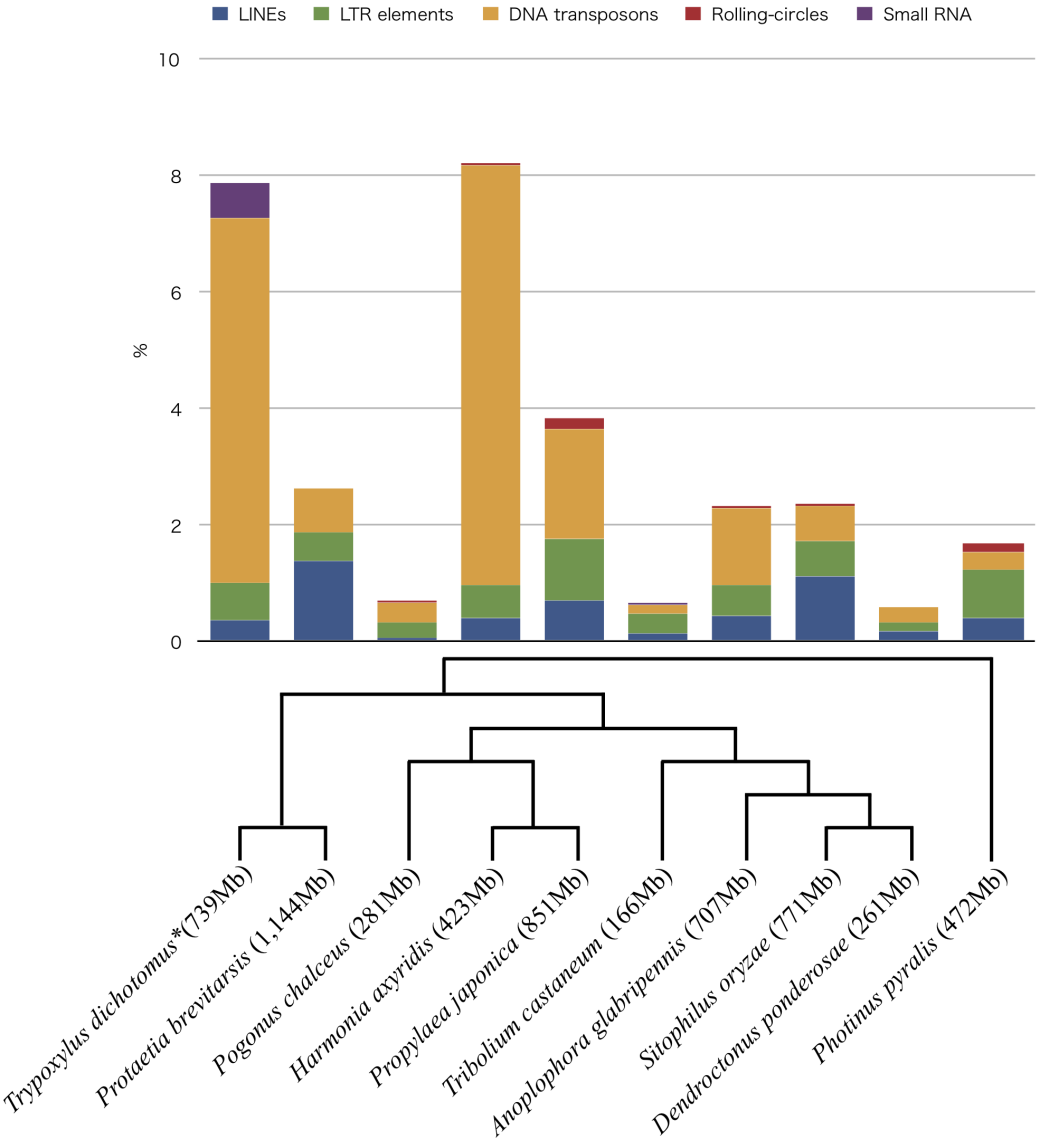
The interspersed repeats and low complexity DNA sequences were screened using RepeatMasker. *Trypoxylus dichotomus* (*decoded in this study)*, Protaetia brevitarsis, Pogonus chalceus, Harmonia axyridis, Propylaea japonica, Tribolium castaneum, Anoplophora glabripennis, Sitophilus oryzae, Dendroctonus ponderosae* and *Photinus pyralis* were compared. The vertical axis of the bar plot shows the percentage of repeat sequences in each genome. The phylogenetic tree is based on previous studies (McKenna *et al.*, 2019 and Majerus *et al.*, 2016).

## Description

Genomic research of the Japanese rhinoceros beetle, *Trypoxylus dichotomus* has been of interest since their large body size (~35g) and sexual dimorphizm in adult horn, and although several transcriptome studies had been conducted (Ogata *et al.*, 2017; Ohde *et al.*, 2018; Warren *et al.*, 2014), the genome itself has not yet been deciphered. The haploid chromosome number, n, 10, and the male heterogamety of XY-type established for the beetle are known (Kudoh *et al.*, 1970). Here we sequenced and assembled the genome of the beetle. Last-instar female larvae were harvested at Fuchu-shi, Tokyo, Japan and reared in leaf mold. Larval fat bodies were isolated. Total DNA was extracted from the fat bodies using the Qiagen Blood & Cell Culture DNA Maxi Kit (Qiagen, Gaithersburg, MD). Isopropanol and the eluted DNA mixture were dispensed to 1.5 mL tube at the step of centrifuging DNA for making pellet. DNA shearing performed using Megaruptor® 3 (Diagenode, Liège, Belgium), targeting an average fragment size of 20 kb. The SMRTbell Express template preparation kit 2.0 (Pacific Biosciences, Menlo Park, CA) and the Barcoded Overhang Adapter Kit (Pacific Biosciences) were used to ligate hairpin adapters required for sequencing to the fragmented DNA. The library was size selected using the SageELF (Sage Science, Beverly, MA) and the 0.75% Agarose DNA ELF cassette with marker 75 (Sage Science). Sequence template was prepared by Sequel Ⅱ Binding Kit 2.0 (Pacific Biosciences) and Sequel Ⅱ DNA Internal Control Kit 1.0 (Pacific Bio-sciences). Sequencing was done on the Sequel Ⅱ System (Pacific Biosciences) using Sequel Ⅱ Sequencing Kit 2.0 (Pacific Biosciences) and Sequel SMRT Cell Oil (Pacific Biosciences). Data processing was performed using SMRT Link v8.0.0 (Pacific Biosciences). In total, 18,816,210,693 bp, 1,363,523 reads were generated as CCS reads. The G+C content of the beetle genome was 35.99%. Library preparation and sequencing were performed at the Takara Bio Inc. (Kusatsu, Shiga, Japan). Reads were trimmed, corrected, and assembled using the Hifiasm software version 0.3.0 (Gheng *et al.*, 2020). The final assembled genome consisted of 739,405,423 bp in 2,347 contigs, with 24.5× mean coverage. The largest contig size was 36,507,117 bp. The N50 of the contigs was 7,929,129 bases. The genome was annotated using public transcriptome data (DRA004723 and DRA006307), hisat2 (Kim *et al.*, 2019) and cufflinks (Trapnell *et al.*, 2010). The mapped rate of sequence data DRA004723 to the genome was 94%. The transcripts number was 34,598. For the genomic sequence, 99.6% of the BUSCO orthologs were detected as complete sequences, 0.1% were fragmented and 0.3% were missing (-l option was insecta_odb10) (Simão *et al.*, 2015). The interspersed repeats and low complexity DNA sequences in the genome were screened using RepeatMasker (Smit *et al.*, 2015). LINEs, LTR elements, DNA transposons, and small RNAs occupied 0.36%, 0.64%, 6.28%, and 0.61% of the genome, respectively. The genome of *Trypoxylus dichotomus* showed more small RNAs than other Coleopteran genomes (*Protaetia brevitarsis* (GCA_004143645.1), *Pogonus chalceus* (GCA_002278615.1), *Harmonia axyridis* (GCA_003402655.1) (Ando *et al.*, 2018), *Propylaea japonica* (GCA_013421045.1) (Zhang *et al.*, 2020), (*Tribolium castaneum* (GCF_000002335.3) (Herndon *et al.*, 2020), *Anoplophora glabripennis* (GCF_000390285.2), *Sitophilus oryzae* (GCF_002938485.1), *Dendroctonus ponderosae* (GCA_000346045.2) (Keeling *et al.*, 2013) and *Photinus pyralis* (GCF_008802855.1) (Fallon *et al.*, 2018)).

## Reagents

This whole-genome shotgun project has been deposited at GenBank under the accession no. BNES00000000.1. Raw reads were deposited at DDBJ SRA under the accession no. DRA010769.
